# TRANSGENIC MOUSE MODELS OF ALZHEIMER’S DISEASE

**DOI:** 10.1093/geroni/igz038.3077

**Published:** 2019-11-08

**Authors:** Charnae A Henry-Smith, Xianlin Han

**Affiliations:** 1 Howard University, Washington, D.C., United States; 2 Sam and Ann Barshop Institute for Longevity and Aging Studies, San Antonio, Texas, United States

## Abstract

Alzheimer’s disease is a progressive brain disease that slowly destroys memory and thinking skills. Alzheimer’s is characterized by an increase in Aβ plaques , and tau tangles. Neurons in the brain have axons covered in myelin sheath that connect microglia and astrocytes. The myelin sheath is composed of about 70% lipid composition; Sulfatide contributing to 30% overall. Sulfatide changes the morphology of primary microglia to their activated form. To study the role of microglia activation and sulfatide levels, three different mouse models were created: APP KI mice, CST Whole Body Ko mice, and cCST (conditional) KO. In order to create the genotype of the APP KI mice, a breeding mouse line was created. The APP KI gene had to be introduced in Plp1-Cre and cCST KO crossed mice to receive a working mouse model. During the duration of breeding for the APP KI mice, a preliminary experiment was performed on the CST KO mice. These mice were given the PLX3397 diet with the aim to remove the microglia and to see the effect of Aβ plaques. The PLX3397 will reduce the microglia targeting the CSF1R. After consuming the diet, the mice were harvested to collect tissues from the brain and spinal cord. Lipidomics and immunohistology were performed. In conclusion, we will continue the breeding of the CST flox/flox / Plp1-Cre / APP KI mice, and the drug dosage and treatment to be used in our APP KI mice will be based on preliminary data from our CST mice.

